# Validation and reproducibility of cardiovascular 4D-flow MRI from two vendors using 2 **×** 2 parallel imaging acceleration in pulsatile flow phantom and in vivo with and without respiratory gating

**DOI:** 10.1177/0284185118784981

**Published:** 2018-06-26

**Authors:** Jelena Bock, Johannes Töger, Sebastian Bidhult, Karin Markenroth Bloch, Per Arvidsson, Mikael Kanski, Håkan Arheden, Frederik Testud, Andreas Greiser, Einar Heiberg, Marcus Carlsson

**Affiliations:** 1 Department of Clinical Sciences, Lund University, Clinical Physiology, Skåne University Hospital, Lund, Sweden; 2 Department of Diagnostic Radiology, Lund University, Skåne University Hospital, Lund, Sweden; 3 Department of Biomedical Engineering, Faculty of Engineering, Lund University, Lund, Sweden; 4 Philips Healthcare, Lund, Sweden; 5 Lund University Bioimaging Center, Lund University, Lund, Sweden; 6 Siemens Healthcare AB, Malmö, Sweden; 7 Siemens Healthcare GmbH, Erlangen, Germany

**Keywords:** 4D-flow, heart failure, valvular regurgitation, cardiac output

## Abstract

**Background:**

4D-flow magnetic resonance imaging (MRI) is increasingly used.

**Purpose:**

To validate 4D-flow sequences in phantom and in vivo, comparing volume flow and kinetic energy (KE) head-to-head, with and without respiratory gating.

**Material and Methods:**

Achieva dStream (Philips Healthcare) and MAGNETOM Aera (Siemens Healthcare) 1.5-T scanners were used. Phantom validation measured pulsatile, three-dimensional flow with 4D-flow MRI and laser particle imaging velocimetry (PIV) as reference standard. Ten healthy participants underwent three cardiac MRI examinations each, consisting of cine-imaging, 2D-flow (aorta, pulmonary artery), and 2 × 2 accelerated 4D-flow with (Resp+) and without (Resp−) respiratory gating. Examinations were acquired consecutively on both scanners and one examination repeated within two weeks. Volume flow in the great vessels was compared between 2D- and 4D-flow. KE were calculated for all time phases and voxels in the left ventricle.

**Results:**

Phantom results showed high accuracy and precision for both scanners. In vivo, higher accuracy and precision (*P* < 0.001) was found for volume flow for the Aera prototype with Resp+ (–3.7 ± 10.4 mL, r = 0.89) compared to the Achieva product sequence (–17.8 ± 18.6 mL, r = 0.56). 4D-flow Resp− on Aera had somewhat larger bias (–9.3 ± 9.6 mL, r = 0.90) compared to Resp+ (*P* = 0.005). KE measurements showed larger differences between scanners on the same day compared to the same scanner at different days.

**Conclusion:**

Sequence-specific in vivo validation of 4D-flow is needed before clinical use. 4D-flow with the Aera prototype sequence with a clinically acceptable acquisition time (<10 min) showed acceptable bias in healthy controls to be considered for clinical use. Intra-individual KE comparisons should use the same sequence.

## Introduction

Quantification of the intracardiac or vascular blood flow in three dimensions over time using magnetic resonance imaging (MRI), so called 4D-flow, enables the study of novel physiological parameters revealing insights into pathophysiological conditions. One example is kinetic energy (KE), which has shown differences between healthy volunteers and patients (1–3) and has been proposed to provide risk stratification of patients with various heart diseases (3). Furthermore, the use of 4D-flow has been proposed as an effective and more accurate method to quantify unstable and spatially complex flow patterns, e.g. in valvular lesions (3–6). Also, one single 4D-flow acquisition may save time compared to several 2D-flows in patients (7–9). Validation of the used techniques are needed in order for 4D-flow to be utilized clinically and to compare results between centers. To this date, most validation studies have been limited to one vendor platform. Thus, there is a need to demonstrate how 4D-flow performs on scanners from different vendors. Furthermore, the robustness of measured physiological parameters, i.e. repeatability, is most often studied using a scan and rescan at the same imaging occasion (10), but to be a reliable biomarker the measured parameter should be reproducible, i.e. stable over a longer time period (10). Finally, the data acquisition needs to be fast enough to fit into a clinical protocol. One way to achieve this is to acquire the images without respiratory navigator gating, which lowers the scanning time by up to 60% (11). The use of non-respiratory-gated 4D-flow in patients and controls with moderate parallel imaging acceleration using acceleration factor 2 has previously been validated (11). Modern scanner hardware allows for higher acceleration factors due to better signal-to-noise ratio, enabling further reduction of scan times by increasing the parallel imaging acceleration factor beyond 2 (12). However, the effect of higher parallel imaging acceleration factors on 4D-flow data quality with and without respiratory gating is not known.

Therefore, the aims of this study were to validate highly accelerated 4D-flow sequences from two vendors head-to-head with respect to flow volumes and KE by performing scans in vitro in a three-dimensional (3D) pulsatile phantom setup with laser particle image velocimetry (PIV) as the reference standard, and in vivo: (i) with and without respiratory gating; (ii) on different platforms on the same day for reproducibility; and (iii) on the same scanner on different days for repeatability.

## Material and Methods

The regional ethical committee approved the study and written informed consent was obtained from each subject. Healthy controls (n = 10, see Table 2) underwent 1.5-T cardiac MRI scans including 2D cine in long and short-axis planes during breath-hold, 2D flow of the aorta and pulmonary artery during free-breathing, and 4D-flow. The scanners used were a MAGNETOM Aera, Siemens Healthcare, Erlangen, Germany and an Achieva dStream, Philips Healthcare, Best, The Netherlands. The study protocol included: (i) repeating the scan back-to-back on the same day with the two different scanners for reproducibility test (10); and (ii) repeating the examination on one of the scanners within two weeks for repeatability (10). To minimize differences caused by circadian rhythm on cardiac physiology, all MRI scans were performed after 13:00 and the order of the two scans alternated between individuals. Additionally, all participants fasted 2 h before the measurements and were provided with a small snack (fruit and pastry) and water before each scan.

Whole heart 4D-flow with retrospective ECG-triggering and 2 × 2 acceleration (GRAPPA and SENSE reconstruction for Aera and Achieva, respectively) were acquired with (Resp+) and without (Resp−) respiratory navigator gating. On the Siemens scanner, a prototype 4D-flow sequence (WIP 785K) enabling retrospective ECG-triggering was used. The software version at Achieva dStream was R5.1.7. Typical parameters for 4D-flow are shown in Table 1. Number of time phases acquired depended on heart rate and was set to the maximum with a preserved segmentation factor/views per segment of 2 and images were then reconstructed to 40 time phases. The images covered the entire heart in an oblique transversal view, with phase encoding (fold-over direction) in the anteroposterior direction.

**Table 1.** 4D-flow sequence parameters in vivo.

4D-flow parameters	Siemens 1.5-T Aera	Philips 1.5-T Achieva
TE (ms)	3.5	2.5
TR (ms)	46	46
Flip angle (°)	8	8
Spatial resolution (mm^3^)	3 × 3 × 3	2.9 × 2.9 × 3
Temporal resolution reconstructed (ms)	22	26
Matrix size	82 × 87 × 61	84 × 84 × 61
VENC (cm/s)	100	100
Parallel imaging (phase × slice)	2 × 2 (GRAPPA)	2 × 2 (SENSE)
Oversampling in slice direction	No	1.4
**2D flow parameters in vivo**		
TE (ms)	2.7	5.4
TR (ms)	9.8	8.7
Flip angle (°)	20	15
Spatial resolution (mm^2^)	1.55 × 1.55	1.25 × 1.25
Temporal resolution acquired (ms)	29	29
VENC (cm/s)	200	200
Parallel imaging	No	No
Product surface coil array	Body 60 with 30 elements in the anterior and posterior parts each	Anterior dS Torso coil with 32 elements and the dS Posterior coils integrated in the scanner table

**Table 2.** Participant characteristics.

Male/Female	7/3
Age (years)	32 ± 8
Height (cm)	180 ± 8
Weight (kg)	77 ± 11
Heart rate (beats per minute)	58 ± 9
EDV (mL)	181 ± 35
ESV (mL)	70 ± 17
EF (%)	61 ± 4

### Image analysis

All MR images were analyzed using the Segment software (http://segment.heiberg.se) with an in-house developed module for 4D-PC-MRI analysis (13). Quality was graded by two observers (with ten and seven years of experience with 4D-flow, respectively) in consensus on a four-grade scale of 0–3, where 0 is excellent quality, 1 is good, 2 is acceptable, and 3 is inadequate image quality warranting exclusion of data. A first-order phase background correction (14,15) and phase unwrapping (16) was performed before analysis.

The integrated flow per heartbeat (flow volume) was calculated from the ascending and descending aorta and pulmonary artery from both 2D- and 4D-flow acquisitions in identical imaging planes. The vessels were semi-automatically outlined in the 2D-flow images and the contours transferred to the 4D-flow. The ratio of pulmonary to aortic (systemic) flow (QP/QS) was calculated as this is a common clinical metric obtained from cardiac MRI.

The endocardium of the right and left ventricles was manually outlined in all time phases of the cine short-axis stack and the delineations transferred to the 4D-flow dataset. KE for each voxel in the ventricle was calculated as KE = ½mv^2^ (m is mass of the voxel and v is the velocity in the voxel) and summed over the ventricle for each time phase.

Stroke volume (SV) from cine images was measured for comparison to flow to determine if 4D-flow underestimates or 2D-flow overestimates SV. Inter-observer variability was determined by a second observer for 66 vessels in 22 4D-flow datasets randomly selected from both scanners.

### In vitro validation

**Fig. 1.**
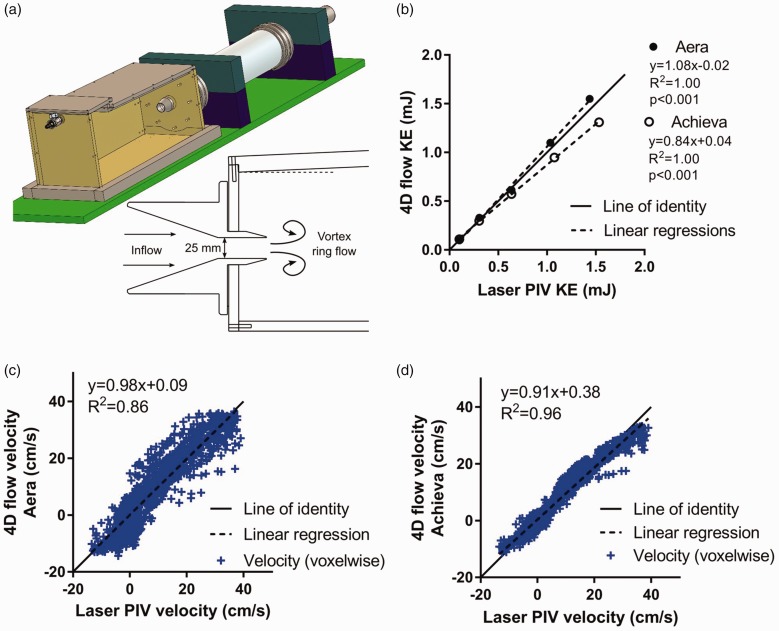
Validation setup and results. (a) Phantom geometry. A custom-built pump was used to produce a pulsatile flow through a nozzle, generating vortex rings downstream from the nozzle orifice. MR 4D-flow was acquired and compared to laser PIV data. (b) Validation of KE for both scanners. Good agreement was shown for Aera and a small underestimation for Achieva. (c, d) Validation of velocity in individual voxels in the sagittal centerline of the flow phantom for (c) Aera and (d) Achieva.

Phantom validation was performed as shown in Fig. 1a. The phantom setup enables measurement of a pulsatile and fully 3D water flow with both 4D-flow MRI and laser PIV as the reference standard. Five different pump programs were used, with SV in the range of 12–37 mL. Acquisition parameters for 4D-flow were similar to the in vivo scans described above (see Supplementary file for more detailed information). Laser PIV resulted in a spatial resolution of 1.5 × 1.5 mm^2^ in a single sagittal slice along the main flow direction and a temporal resolution of 10 ms. Gadolinium contrast agent (Dotarem, Guerbet, France) was added to the water to achieve a T1 relaxation time roughly comparable to that of blood (1350–1550 ms). Pump stability was quantified by measuring SV before and after each 4D-flow acquisition using the timer and beaker method.

Linear background phase correction was performed for all phantom 4D flow datasets. A linear fit was performed in stationary areas of the phantom in each dataset and the fit then subtracted. Comparison between 4D-flow and laser PIV data was performed on the basis of velocity values along the main vortex flow direction (feet-head) and by computing KE in the vortex ring (17).

### Statistical analysis

Statistical analysis was performed using GraphPad (v5, La Jolla, CA, USA). Values are presented as means ± standard deviation (SD). Differences in results were assessed using the non-parametric paired Wilcoxon test. Results with a *P* value < 0.05 were considered statistically significant. Pearson’s correlation analysis and Bland–Altman analysis were performed and presented as bias ± SD.

## Results

### In vitro phantom validation

Voxel-wise comparison of velocities between 4D-flow and laser PIV showed a strong correlation and low bias for both Aera (y = 0.98x + 0.09, slope 95% confidence interval [CI] = 0.96–1.00, R^2 ^= 0.86, bias = –0.01 ± 4.54 cm/s) and Achieva (y = 0.91x + 0.38, slope 95% CI = 0.90–0.92, R^2 ^= 0.96, bias = –0.11 ± 2.28 cm/s) scanners. SV on 4D-flow and 2D-flow showed strong correlation and very low bias on both Aera (y = 1.03x–0.54, R^2 ^= 0.99, bias =0.06 ± 0.98 mL) and Achieva (y = 1.01x–0.01, R^2 ^= 1.00, bias = 0.18 ± 0.37 mL). SV had similar correlation and agreement with timer and beaker measurements on Aera (y = 1.1x–3.3, R^2 ^= 0.99, bias = −0.14 ± 1.75 mL) and Achieva (y = 1.0x–0.3, R^2 ^= 0.99, bias = –0.54 ± 0.97 mL). KE showed good agreement compared to laser PIV on Aera (y = 1.08x–0.02, R^2 ^= 1.00, bias =0.04 ± 0.05 mJ) and a slight underestimation on Achieva (–y = 0.84x + 0.04, R^2 ^= 1.00, bias = 0.08 ± 0.10 mJ). Results are summarized in Fig. 1b–d.

Pump stability was excellent (Achieva: difference after-before 4D: –0.1 ± 0.3 mL, y = 1.00x–0.14, R^2 ^= 1.00, Aera: difference = 0.0 ± 0.1 mL, y = 1.01x–0.09, R^2 ^= 1.00), and pump SV showed a small difference between scanners (0.4 ± 0.4 mL, y = 1.02x–0.13, R^2 ^= 1.00).

### In vivo validation

In total, 57 4D-flow scans were acquired in the ten individuals (characteristics summarized in Table 2). One participant could not undergo repeated MR within 14 days due to logistical reasons and two acquisitions were therefore not performed; in addition, one acquisition (Achieva) did not reconstruct correctly and images were therefore lost. The rescans were performed 6 ± 3 days after the first examination.

**Fig. 2.**
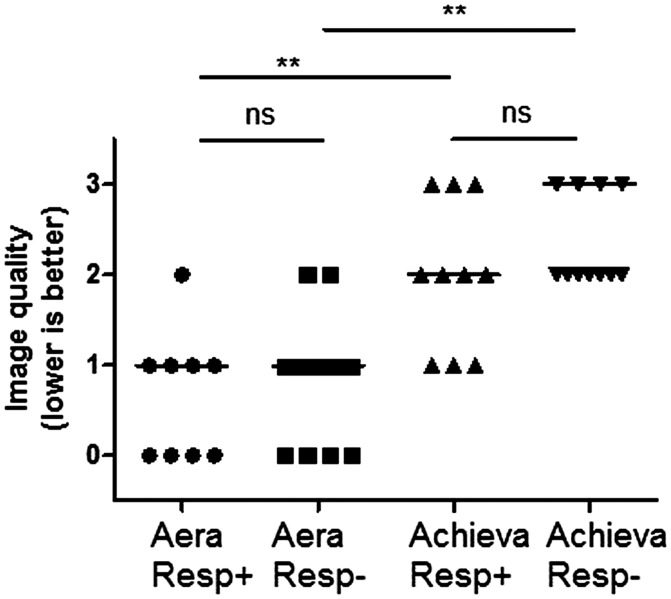
Image quality assessment of 4D-flow data. Grading scale: 0 = excellent image quality to 3 = inadequate image quality. Lines show median image quality. **P < 0.01.

Average scan duration for Aera was 9 ± 3 (Resp+) and 6 ± 2 (Resp−) min, and for Achieva 17 ± 3 (Resp+) and 10 ± 1 (Resp−) min. Image quality was better for Aera compared to Achieva for both respiratory (0.7 ± 0.6 vs. 2.0 ± 0.7, lower means better image quality) and non-respiratory-gated (0.9 ± 0.6 vs. 2.5 ± 0.5) acquisitions. Intra-scanner comparison showed no difference in image quality between respiratory and non-respiratory triggered acquisitions (Fig. 2). On the Aera, no 4D-flow acquisitions had inadequate image quality but 4 Resp+ (31%) and 8 Resp− (57%) 4D-flow acquisitions had inadequate image quality on the Achieva. Data are presented for all acquired data and with exclusion of acquisitions with inadequate image quality.

**Fig. 3.**
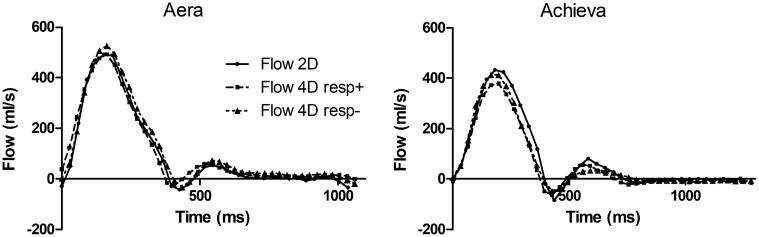
Pulmonary artery flow curves from 2D-flow and 4D-flow with (Resp+) and without (Resp−) respiratory gating on the two scanners at the same day, same participant.

**Fig. 4.**
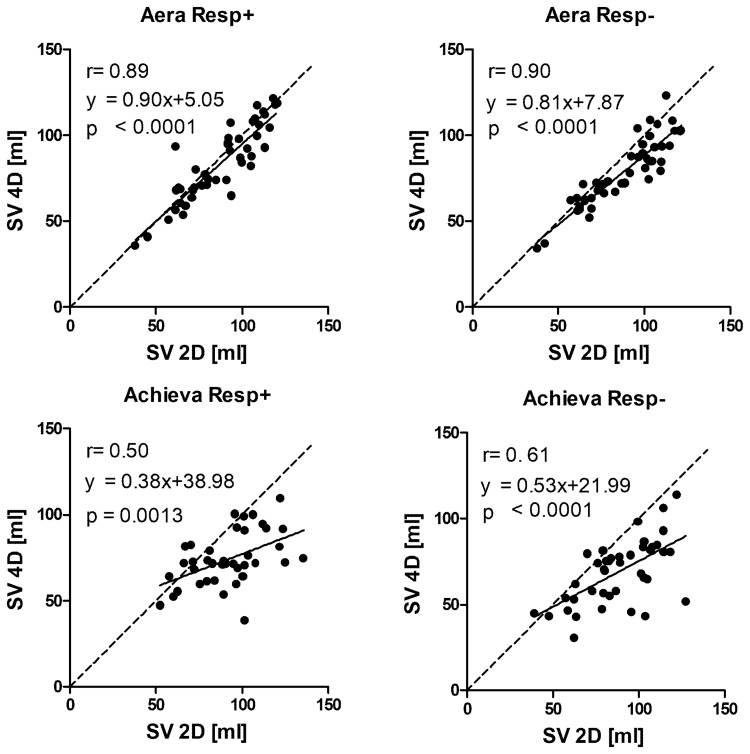
Correlation between SV from 4D-flow and 2D-flow acquisitions. Line of identity is shown by dashed line and line of regression with solid line.

**Fig. 5.**
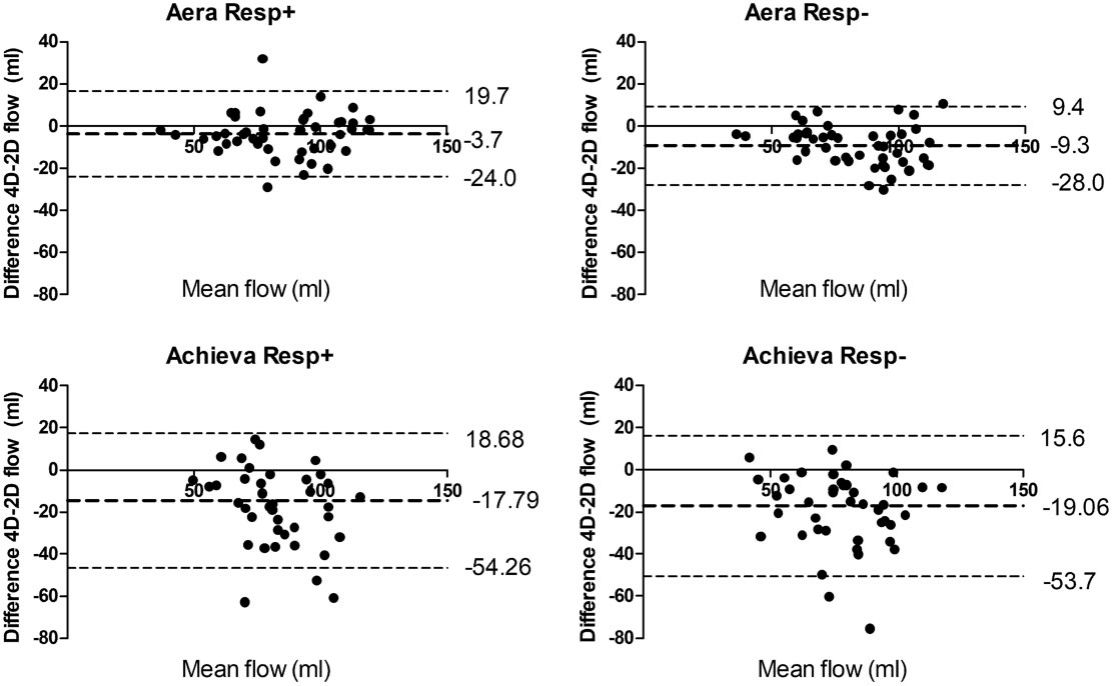
Bland–Altman analysis of 4D-flow vs. 2D-flow volumes.

Examples of flow curves from one individual are shown in Fig. 3. Compared to standard 2D-flow, 4D-flow underestimated the flow volume on both Aera and Achieva (Table 3, Figs. 4 and 5) scanners. 4D-flow scans with respiratory gating on Aera showed the lowest bias of all scans and had higher precision both compared to Aera 4D-flow Resp− (*P* = 0.005) and Achieva Resp+(*P* < 0.001). Bias was not different for Resp+ 4D-flow compared to Resp− on Achieva (*P* = 0.87). The QP/QS was slightly higher for 4D-flow compared to 2D-flow for both scanners (Table 3). The use of respiratory gating did not change the QP/QS for Aera (*P* = 0.89) or Achieva (*P* = 0.057).

**Table 3.** Bias of flow volumes and the ratio of pulmonary and aortic flow (QP/QS) on 4D-flow vs. 2D-flow acquisitions for all acquired data and when excluding scans with inadequate image quality on Achieva.

	Aera 1.5-T (n = 15)	Achieva 1.5-T (n = 15)	Achieva 1.5-T (exclusion of scans with inadequate image quality)
Bias 4D Resp+ and 2D mL (%)	−3.7 ± 10.4 (−4.7 ± 12.5)*^†^	−17.8 ± 18.6 (−20.9 ± 22.2)	−16.3 ± 18.6 (−18.0 ± 20.1) (n = 9)
Bias 4D Resp− and 2D mL (%)	−9.3 ± 9.6 (−10.9 ± 10.5)^‡^	−19.1 ± 17.7 (−24.5 ± 23.2)	−21.2 ± 22.2 (−26.0 ± 26.5) (n = 6)
QP/QS 2D (range)	1.01 ± 0.07 (0.91–1.16)	1.04 ± 0.08 (0.91–1.18)	1.03 ± 0.07 (0.91–1.12) (n = 9)
QP/QS 4D Resp+ (range)	1.10 ± 0.13 (0.91–1.32)	1.19 ± 0.28 (0.90–1.86)	1.13 ± 0.20 (1.00–1.53) (n = 9)
QP/QS 4D Resp− (range)	1.10 ± 0.11 (0.90–1.26)	1.32 ± 0.32 (0.97–2.27)	1.46 ± 0.44 (0.99–2.27) (n = 6)

* *P* < 0.01 for Resp+ vs. Resp−.

† *P* < 0.01.

‡ *P* < 0.001, for Aera vs. Achieva.

**Fig. 6.**
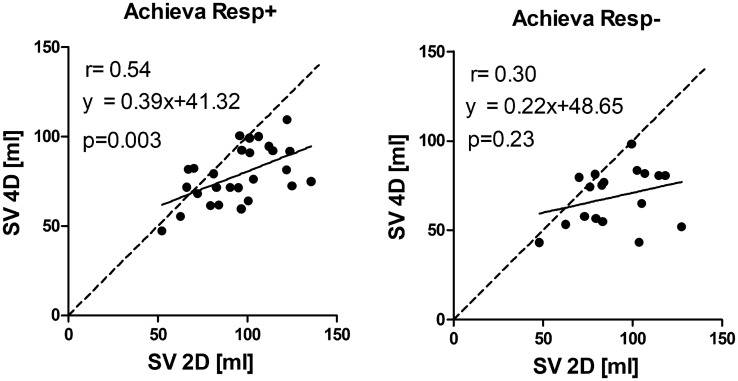
Correlation between SV from 4D-flow and 2D-flow acquisitions from Achieva when excluding acquisitions with suboptimal image quality. Line of identity is shown by dashed line and line of regression with solid line.

SV from 2D-flow before and after the 4D-flow acquisitions showed low bias and variability (0.6 ± 8.0 mL), which indicates that there was no systematic physiological drift in SV during scans.

Inter-observer variability for 4D-flow measurements (n = 66 vessels) was 1.3 ± 2.1 mL (1.5 ± 2.5%) and showed a very strong correlation (r = 0.99, y = 1.04x–2.2 mL). The inter-observer variability for 2D-flow was 0.2 ± 5.9 mL.

KE curves over the heart cycle with in vivo 4D-flow showed that late diastolic peak KE and mean KE were lower on the Aera compared to the Achieva (Table 4) when using navigator respiratory gating (Resp+). The use of respiratory gating was associated with lower peak KE in systole and late diastole for Aera but higher systolic and lower early diastolic peak KE for Achieva (Table 4).

**Table 4.** Kinetic energy (KE): for Achieva, results are given both for all scans (middle two columns) and for scans when excluding inadequate image quality (right two columns).

KE	Aera Resp+ (n = 15)	Aera Resp− (n = 15)	Achieva Resp+ (n = 14)	Achieva Resp− (n = 13)	Achieva Resp+ (n = 10)	Achieva Resp− (n = 6)
Peak KE systole (mJ)	3.9 ± 1.5*	4.2 ± 1.7	4.7 ± 1.4*	4.0 ± 1.4	4.6 ± 1.6	4.1 ± 1.3
Peak KE early diastole (mJ)	5.3 ± 1.9	5.6 ± 2.3	5.6 ± 1.6*	5.9 ± 1.8	5.0 ± 1.1	5.8 ± 2.7
Peak KE late diastole (mJ)	1.4 ± 0.6^†,^**^‡^**	1.8 ± 0.9	2.2 ± 0.8	2.2 ± 0.8	2.1 ± 0.7	1.9 ± 0.6
Mean KE (mJ)	1.7 ± 0.6**^§^**	1.9 ± 0.8	2.3 ± 0.5	2.2 ± 0.6	2.1 ± 0.4	2.2 ± 0.6

* *P* < 0.05.

† *P* < 0.001 for Resp+ vs. Resp−.

‡ *P* < 0.05.

§ *P* < 0.01, for Aera vs. Achieva.

The repeatability of KE for both scanners is shown in Table 5. Notably, the bias for a 4D-flow acquisition compared to a repeated acquisition within 14 days on the same scanner was generally lower compared to the bias comparing scans from the two vendors on the same day.

**Table 5.** Reproducibility shown as bias ± SD between different days on the same scanner (left columns, scan vs. rescan) and repeatability as bias ± SD between scanners the same day (right columns, Aera vs. Achieva).

KE (all scans)	KE Resp+ scan vs. rescan (n = 9)	KE Resp− scan vs. rescan (n = 9)	KE Resp+ Aera vs. Achieva (n = 10)	KE Resp− Aera vs. Achieva (n = 9)
Peak KE systole (mJ)	−0.29 ± 1.06	−0.51 ± 0.74	−0.91 ± 1.29	−0.01 ± 1.69
Peak KE early diastole (mJ)	−0.79 ± 1.48	−0.82 ± 1.98	−0.34 ± 1.50	−0.03 ± 2.29
Peak KE late diastole (mJ)	0.16 ± 0.39	0.01 ± 0.70	0.80 ± 0.56	0.80 ± 0.56
Mean KE (mJ)	−0.10 ± 0.40	−0.21 ± 0.45	−0.64 ± 0.46	−0.33 ± 0.83
KE (scans with inadequate quality excluded)	KE Resp+ scan vs. rescan (n = 7)	KE Resp− scan vs. rescan (n = 6)	KE Resp+ Aera vs. Achieva (n = 8)	KE Resp− Aera vs. Achieva (n = 4)
Peak KE systole (mJ)	−0.53 ± 0.92	−0.50 ± 0.52	−1.15 ± 1.33	−0.42 ± 0.25
Peak KE early diastole (mJ)	−0.50 ± 1.32	−0.20 ± 2.1	−0.39 ± 1.5	−1.04 ± 1.22
Peak KE late diastole (mJ)	0.00 ± 0.14	0.20 ± 0.64	−0.75 ± 0.63	−0.22 ± 0.60
Mean KE (mJ)	−0.15 ± 0.31	−0.04 ± 0.35	−0.57 ± 0.50	−0.42 ± 0.25

KE, kinetic energy.

## Discussion

This study validated 4D-flow sequences with high acceleration from two different vendors’ MR scanners head-to-head showing differences in accuracy and precision of flow measurements between vendors in vivo. Although in vitro phantom experiments showed high accuracy and precision for both scanners, in vivo scans demonstrated significantly higher accuracy and precision for volume flow on the Aera 4D-flow sequence compared to the Achieva. This highlights the difficulties in translating good in vitro results to in vivo measurements and highlights the importance of rigorous validation at each site before clinical use. The flow results from the 4D-flow prototype sequence show the possibility for 4D-flow to replace repeated 2D-flow measurements. Furthermore, this study showed the possible use of free breathing 4D-flow acquisitions without respiratory gating with high acceleration on the Aera with the prototype sequence with low differences in bias compared to results from respiratory gating and thus a further possibility to decrease 4D-flow scan time. KE measurements show differences between scanners on the same day that are larger than the differences in KE from the same scanner over time. This means that longitudinal KE measurements need to be performed using the same MR scanner.

A previous multi-center, multi-vendor 2D-flow validation showed that velocity offset varied between sites having the same vendor and sequence (18). This may also apply to 4D-flow and the suboptimal performance of 4D-flow on the Achieva scanner in this study may be scanner- or software-specific rather than vendor-specific. However, of note, a recent 4D-flow two-center validation study performed on Philips Ingenia scanners showed similar correlation between aortic and mitral flows as in our study (19). Until a multi-center, multi-vendor 4D-flow validation study has shown agreement between scanners, validation at each site performing 4D-flow is therefore needed before clinical or research use of this technique. The proposed validation in this study compared 2D-flows in the aorta and pulmonary artery. The calculated QP/QS was in the range of 0.9–1.3, which is deemed to be acceptable for clinical use for shunt quantifications.

### Relation to earlier studies

Our results on the Philips Achieva dStream show similar correlations as Garg et al. using a 1.5-T Philips Ingenia without respiratory navigating using a spoiled gradient echo sequence with k-segmentation of 2 and SENSE 2 in the phase encoding plane (19). Garg et al. found a modest correlation for net-flow consistency between aortic outflow and mitral inflow (r = 0.58, y = 0.74x + 26.99) similar to our results comparing 4D-flows to 2D-flows (r = 0.51, y = 0.53x + 21.99). They concluded that the 4D-flow using EPI-acceleration showed better image quality and higher consistency compared to the SENSE-accelerated sequence, however only with a modest correlation to 2D-flow for velocities. Previous work showed better accuracy on 4D-flow on Philips Achieva 3-T compared to 1.5-T Achieva with SENSE-acceleration of 2 and k-segmentation 2 and that acceleration with k-t-BLAST causes underestimation of flow volumes (12). This underestimation of velocity and flow using k-t-BLAST was also replicated in the study by Garg et al. (19) but not seen in the 3-T study using a 32-channel coil (20). We have previously shown a low bias of 4D-flow with SENSE-acceleration of 2 and k-segmentation 2, without respiratory gating validating the mitral inflows against planimetric SV in healthy volunteers and patients (11), and the Resp− results of this study reinforce this view.

Kamphuis et al. recently evaluated scan–rescan SV using an EPI-accelerated 4D-flow sequence compared to planimetric SV on a 3-T Philips Ingenia system (21) and found lower SV from 4D-flow but good 4D in-scan consistency. Compared to our study with comparison of 2D-flow and 4D-flow on a 1.5-T Philips scanner, Kamphuis et al. found lower bias (bias on first scan: –11 mL, 95% CI = -34–-12 mL) on the 3-T system. Notably, the bias from Kamphuis et al. are similar to our results from Siemens Aera 1.5T without respiratory navigator. Petersson et al. showed lower correlation between aortic and pulmonary flows with cartesian 4D-flow (SENSE 2 and k-segmentation 2, r = 0.73, y = 0.79x + 24.55) compared to a spiral readout 4D-flow sequence (r = 0.94, y = 1.03 + 3.56) on a 1.5-T Philips Achieva (22). Of note, the correlation in their study was calculated between two flows obtained from 4D-flows and not compared to an external reference such as 2D-flow or planimetric SV.

The bias in our study is lower compared to Hanneman et al. who compared 2D- and 4D-flow for QP/QS in patients with a prospectively triggered 4D-flow sequence in a 1.5-T Siemens Avanto (23). They found bias of –21.9 ± 12.2 mL for the pulmonary artery and –10.7 ± 13.1 mL for aorta; the lower bias in our study may be due to healthy volunteers scanned but may also be due to improvements of the 4D-flow prototype. Valverde et al. validated 4D-flow during free breathing with two-fold SENSE acceleration in patients with Fontan physiology to 2D-flow and showed bias low enough to motivate 4D-flow to replace the longer scan times of repeated 2D-flow acquisitions (9).

Frydrychowicz et al. compared blood-pool contrast-enhanced radially under-sampled PC- 4D-flow (VIPR) to 2D-flow and cine SV on a 3-T GE scanner and found that phantom data correction was necessary to reach accuracy needed for clinical scans (24). Of note, the bias in their study was comparable to our study at the 1.5-T Aera.

Recently, Stoll et al. showed test–retest variability of KE at a 3-T Siemens Trio and showed a higher coefficient of variation for KE at two different time points (6.2–16.1%) compared to scan–rescan at the same day (3.5–17.7%) (25).

In summary, our results using the prototype sequence on the Aera scanner compare favorably to previously published results, and the results without respiratory navigation compare well to results from 3-T scans. The results from the Philips sequence with Cartesian k-space acquisition and SENSE acceleration of 4 are comparable to previously published results with lower acceleration factors but the number of scans with inadequate image quality and the rather large bias should provide caution to use as high acceleration factors as in our study.

The Philips sequence has shown high accuracy and precision in flow phantoms, both for two-fold SENSE acceleration in a previous study (26) and for 2 × 2 SENSE in the present results. However, the in vivo results using 2 × 2 SENSE showed an underestimation of SV compared to 2D-flow. Possible explanations for this discrepancy may include the simplified, symmetric geometry in the phantom compared to human anatomy, an absence of respiratory movement, and less pronounced background phase offsets introduced by the phantom compared to those of the human body.

4D-flow tends to underestimate peak flow velocities due to the lower temporal resolution compared to 2D-flow. This could give lower peak flows as seen in Fig. 4 for Achieva. The phantom validation was only done up to 40 cm/s but the correlation analysis showed that the Achieve had a slope of 0.91, meaning that even velocities as low as 40 cm/s tended to be underestimated. The Aera had a slope of 0.98, meaning that there was little underestimation in this velocity range. Thus, there was no underestimation of KE with the Aera. Further validation studies of 4D-flow are needed to show to what degree higher peak velocities in patients are underestimated. These studies should probably be done in comparison with Doppler ultrasound as 2D-flow MRI may also have problems in detecting the peak velocity in a stenosis, mainly due to the problem of finding the correct imaging plane for detecting the peak velocity with 2D techniques. Indeed, Jarvis et al. showed that 4D-flow showed higher peak velocities compared to 2D-flow but no difference between 4D-flow and Doppler ultrasound (27).

This study included a relatively small population and did not include patients. The reason for not including patients are that three repeated scans with two 4D-flow acquisitions each was used in our study protocol and this is neither feasible nor ethical to ask of patients.

In conclusion, intracardiac 4D-flow accelerated by 2 × 2 parallel imaging could be acquired with adequate quality even without respiratory gating and within a clinically feasible time window (10 min) using a retrospectively ECG-gated prototype 4D-flow sequence on the Aera. Even though in vitro phantom validation showed high accuracy of velocities and KE on both scanners, the in vivo accuracy differed significantly. Therefore, this study shows the importance of in vivo validation of 4D-flow for each scanner and sequence before clinical use. Patients followed over time with KE estimation need to be examined using the same scanner.

## Supplementary Material

Supplementary material
