# Supplementary material for: Validation and reproducibility of cardiovascular 4D-flow MRI from two vendors using 2 × 2 parallel imaging acceleration in pulsatile flow phantom and in vivo with and without respiratory gating
Source: Acta Radiol. 2018 Jun 26;60(3):327–37. doi: 10.1177/0284185118784981 (PMC6402051; doi:10.1177/0284185118784981)
Supplement: Supplementary material [file Supplemental_Material.pdf]

Supplementary file. **4D-flow parameters in phantom**

**4D-flow parameters**	**Siemens Aera 1.5T**	**Philips Achieva 1.5T Achieva**
TE (ms)	3.48	2.38
TR (ms)	5.71	4.07
Flip angle °	8	8
Spatial resolution (mm)	3x3x3 mm	3x3x3 mm
Temporal resolution acquired (ms)	46 ms	32 ms
Matrix size	96x80x52	96x80x52
Slice oversampling	15.4% (to avoid GRAPPA artifact)	-
Parallel imaging (phase-encode direction × slice direction)	2×2 (GRAPPA)	2×2 (SENSE)
Trigger	LED/PPU	ECG simulator (Fogg M311)
